# A Novel Porcine Circovirus-Like Agent P1 Is Associated with Wasting Syndromes in Pigs

**DOI:** 10.1371/journal.pone.0041565

**Published:** 2012-08-24

**Authors:** Libin Wen, Kongwang He, Qi Xiao, Zhengyu Yu, Aihua Mao, Yanxiu Ni, Xuehan Zhang, Bin Li, Xiaomin Wang, Rongli Guo, Junming Zhou, Lixin LV, Jieyuan Jiang

**Affiliations:** Institute of Veterinary Medicine, Jiangsu Academy of Agricultural Sciences·Key Laboratory of Veterinary Biological Engineering and Technology, Ministry of Agriculture·National Center for Engineering Research of Veterinary Bio-products, Nanjing, China; Texas Veterinary Medical Diagnostic Laboratory-Amarillo, United States of America

## Abstract

A novel porcine pathogen tentatively named P1, which was obtained from the sera of the pigs exhibiting clinical signs of postweaning multisystemic wasting syndrome (PMWS) experimentally caused the classical clinic signs and pathologic lesions of the disease in pigs by direct *in vivo* injection with P1 DNA plasmids. Twenty colostrum-fed (CF) pigs that were free of PCV2 and P1 at 1 month of age were randomly designated equally to two groups. Group 1 pigs were each injected with 400 µg of the cloned P1 plasmid DNA into the superficial inguinal lymph nodes and Group 2 were injected with same amount of the empty pSK vector DNA and served as controls. Viremias were positively detected in 8 of 10 P1 infected pigs from 14–21 days post-inoculation (dpi). The 8 infected animals showed pallor of skin and diarrhea. Gross lesions in the pigs euthanized on 35 dpi were similarly characterized by encephalemia, haemorrhage of the bladder mucosa, haemorrhage of the superficial inguinal lymph nodes, lung atrophy and haemorrhage. Histopathological lesions were arteriectasis and telangiectasia of the cavitas subarachnoidealis, interstitial pneumonia, mild atrophy of the cardiac muscle cells, histiocytic hyperplasia of the follicles in the tonsils, and haemorrhage of the inguinal lymph nodes. P1 DNA and antigens were confirmed by PCR and immunohistochemistry in the tissues and organs of the infected pigs, including the pancreas, bladders, testicles/ovaries, brains, lungs and liver. There were no obvious clinical signs and pathological lesions in the control pigs. This study demonstrated that P1 infection is one of the important pathologic agents on pig farms.

## Introduction

Post-weaning multisystemic wasting syndrome (PMWS), an emerging wasting syndrome in pigs first described in 1991 [1]–[3], usually affects pigs between 7 weeks and 15 weeks of age [4]. Although the wasting and respiratory syndrome fits a proportion of late nursery pigs, most of the clinical PMWS signs are variable and non-specific. Most of the syndrome usually includes progressive weight loss, dyspnea, enlargement of the superficial inguinal lymph nodes, and, sometimes, anemia, diarrhea, and jaundice [2], [4]. Coughing, pyrexia, central nervous signs, and sudden death have been occasionally reported [5]. Morbidity may vary from 1% to 2% and up to 30% in complicated cases and the mortality of the sick is up to 80%.

The histopathological lesions of PMWS include interstitial pneumonia, lymphocyte depletion and granulomatous inflammation of the lymphoid tissues, hepatitis and nephritis [3], [6]. PMWS has now been recognized in pigs in the American countries [1], [2], [6]–[10,], many European countries [6], [11]–[22], and some countries in Asia [23]–[25] since it was first identified in Canada. Porcine circovirus type 2 (PCV2) has also been associated with a number of pathological conditions of pigs, such as porcine dermatitis and nephropathy syndrome, reproductive failure, porcine respiratory disease complex, and proliferative and necrotizing pneumonia [26]–[30]. Therefore, the diseases associated with PCV2 infection have become major and the complicated problems have serious economic impact on the swine industry worldwide.

PCV2 has been considered to be the primary causative agent of PMWS. PCV, a small, non-enveloped, spherical virus that contains a single-stranded circular DNA genome of about 1.76 kb [31], is a member of the family of *Circoviridae* [32], which includes two genuses, *Circovirus* and *Gyrovirus*. PCV1 was originally isolated as a contaminant of a continuous porcine kidney cell line (PK-15) [31], [33]. Although the presence of antibodies reactive with PCV1 antigens has been reported in various animal species, including pigs, humans, mice and cattle [11], [34]–[37], PCV1 infection has not produced any clinical disease and, thus, this virus derived from the contaminated PK-15 cell line is considered non-pathogenic [38], [39]. PCV2, however, which showed high levels of nucleotide similarity to PCV1, has been associated with several diseases in pigs, mainly PMWS. Although PCV2 has been shown to be the causal agent for PMWS in both naturally acquired and experimental inoculation of gnotobiotic pigs or conventional pigs with tissue homogenates or molecular DNA clones of PCV2 [27], [40]–[42], a number of experimental studies have found that PCV2 infection alone was not sufficient to induce clinical PMWS, and only resulted in asymptomatic infection with mild to moderate histopathologic lesions [43]–[46]. That indicated that other factors or agents are necessary for a full spectrum of PMWS. We obtained a novel agent (termed P1) from porcine sera by PCR during PCV2 diagnostic work. The porcine serum samples were collected from unthrifty pigs (from 1 month to 3 months of age) exhibiting clinical signs and multisystemic lesions characteristic of PMWS in Hebei, Shandong and Jiangsu provinces and the Beijing municipality of China during 2002–2006. The P1 agent was identified by PCR from two pig farms in Hebei and Jiangsu. Subsequent studies have shown that P1 has became increasingly common on Chinese pig farms [47]. The complete genome of P1 is circular and 648 nucleotides (nt) in size, and the partial genome sequences of P1 have been submitted to GenBank with an accession number of EF514716. A sequence blast indicated that the 5′-terminal 22 nucleotides of P1, ggatccactagtaacggccgcc, might have originated from porcine endogenous retroviruses and the rest of the sequence shared 98.42% identity with the open reading frame 2 (ORF2) of the PCV2 genome (AF381175). Theoretical analysis revealed that the genome of P1 has three potential ORFs [48]. In order to acquire biologically pure agent P1 for pathogenesis studies, we rescued P1 to avoid interference from other swine agents existing in the serum by constructing the P1 molecular DNA clone. The main objectives of the present study were to determine if a molecular DNA clone of P1 might be infectious when transfected into PK-15 cells and injected directly into the superficial inguinal lymph nodes of pigs.

## Results

### The P1 molecular DNA clone is infectious *in vitro*

**Figure 1:**
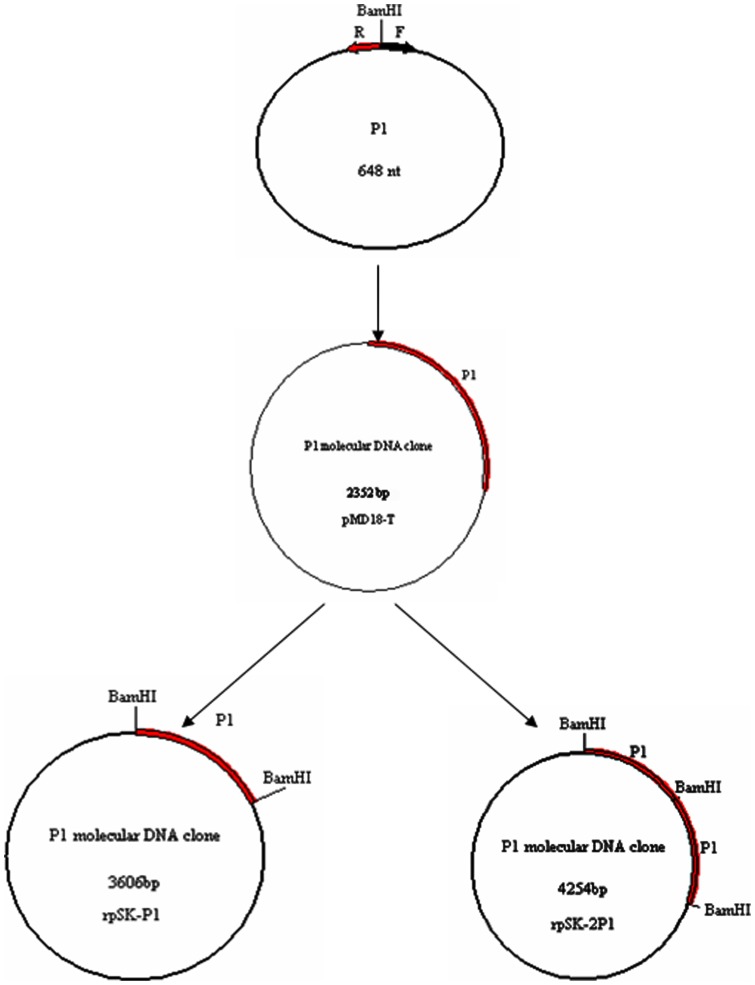
Schematic diagram of the P1 molecular DNA clones constructed.

**Figure 2:**
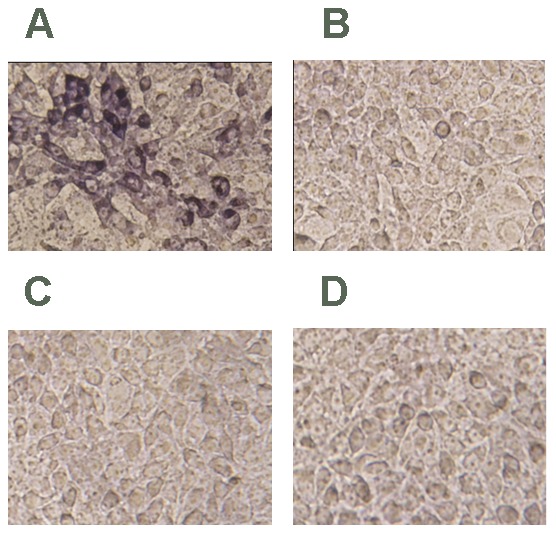
Immunochemical staining of PK15 cells transfected with rpSK-2P1. (A) or rpSK-P1 (B) or empty pSK (C) or untransfected cells were used (D). The presence of P1 antigen is indicated by blue-purple staining.

To construct the P1 molecular DNA clone, a single copy and two copies of the P1 complete genome were ligated into the pSK vector and ligated in tandem into the pSK vector (named rpSK-P1 and rpSK-2P1, respectively), respectively (Figure 1). The infectivity of the P1 molecular DNA clone was determined by in vitro transfection of the PK-15 cells. Immunohistochemistry examination indicated that only PK-15 cells transfected with rpSK-2P1 showed viral antigens. Although a few cells showed nuclear staining, an abundant number of cells showed intense cytoplasmic staining. Untransfected cells and cells mock transfected with rpSK-P1 or empty pSK vector did not exhibit any staining (Figure 2). Thus, we used the rpSK-2P1 for the i*n vivo* transfection experiments in the following study.

### Load of the cell-associated and fluid-phase virus

**Figure 3:**
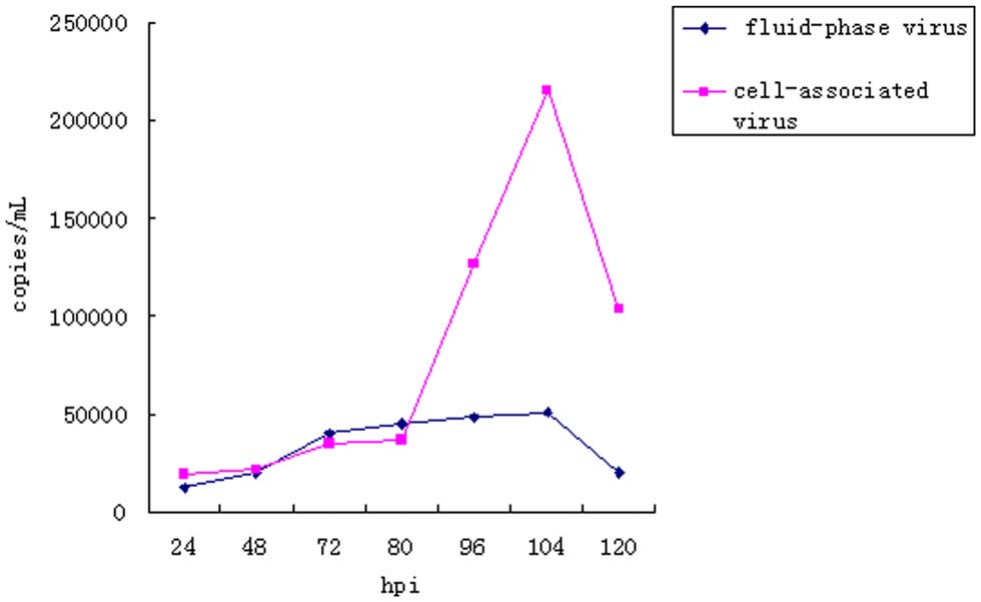
Growth curves of P1 virus in PK-15 cells. Intracellular virus (▪) and extracellular virus(⧫).

The load of the cell-associated and fluid-phase virus is shown in Figure 3. The cell-associated virus slowly increased between 24 hpi and 80 hpi, and then the amount of virus increased more rapidly and reached a maximum titer of about 2×10^5^ copies/mL from 96 hpi to 120 hpi.

No significant change in fluid-phase virus load was observed throughout the experiments. Approximately 10^4^ copies/mL of the virus were found in the fluid-phase materials.

### Electron microscopy observations

**Figure 4:**
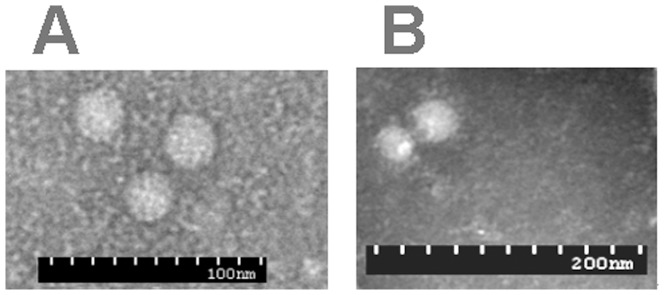
Electron micrographs of P1 particles, obtained from CsCl density gradients, and negatively stained with phosphotungstic acid. (A) Without addition of antiserum. Note the virus particles dispersed at random. (B) Exposure to anti-P1 antiserum generated in rabbit: antibody bridges link the P1 particles.

The non-enveloped viral particles were observed in negatively stained samples obtained by CsCl density gradient centrifugation. The virion was round, approximately 25 nm in diameter by EM. The specificity of the shapes of viruses was demonstrated by immunoelectron microscopy. After admixture of antiserum, the virus particles were predominantly aggregated into clusters. Antibody bridge and antibody coat were found in some particles (Figure 4).

### Viremia

The collected serum samples from all control and inoculated animals at 0 dpi, 7 dpi, 14 dpi, 21 dpi, 28 dpi, and 35 dpi were assayed for P1 viremia by detection of P1 DNA.

Four of 10 pigs at 14 dpi and another 4 of 10 pigs at 21 dpi, and 8 of 10 pigs by 35 dpi from Group 1 were positive for P1 DNA. Viremia was transitory and lasted no more than a week. In contrast, viremia was not detected in the Group 2 control pigs at any dpi (Table 1). PCR products amplified from selected pigs were sequenced. The sequence of the PCR products was identical to the corresponding sequence originating from the P1 molecular DNA clone (data not shown).

**Table 1:** Detection of viremia (P1 DNA) by PCR in sera of infected and control pigs.

Group Inoculum Route of inoculation			dpi						Total positive rate
			0	7	14	21	28	35	
1	P1DNA	Intralymphoid	0/10	0/10	4/10	4/10	0/10	0/10	80%
2	pSK vector	Intralymphoid	0/10	0/10	0/10	0/10	0/10	0/10	0%

All infected and control pigs were negative for PCV2, PRRSV, CSFV, and PRV antibodies from 0 dpi to 35 dpi. Meanwhile, no seroconversion to PPV antibody was detected in any of the 20 pigs and none of the pigs had seroconverted to mycoplasmas antibodies. Based on an ELISA method, serum antibodies to PRRSV, CSFV and PRV were detected by a commercial kit (IDEXX Laboratories, Westbrook, Maine) and to PCV2 with the purified recombinant ORF2 protein expressed in *Escherichia coli*. Serum antibodies to PPV and mycoplasmas were assayed using hemaglutination inhibition assay.

No PCV1, PCV2, PRRSV, CSFV, PRV, PPV, and mycoplasmas viremia were detected from any pig sera at 0 dpi, 7 dpi, 14 dpi, 21 dpi, 28 dpi, and 35 dpi with PCR or RT-PCR assay. These results further indicated that the pigs were not infected by PCV1, PCV2, PRRSV, CSFV, PRV, PPV, and mycoplasmas throughout the study.

### Clinical signs

**Figure 5:**
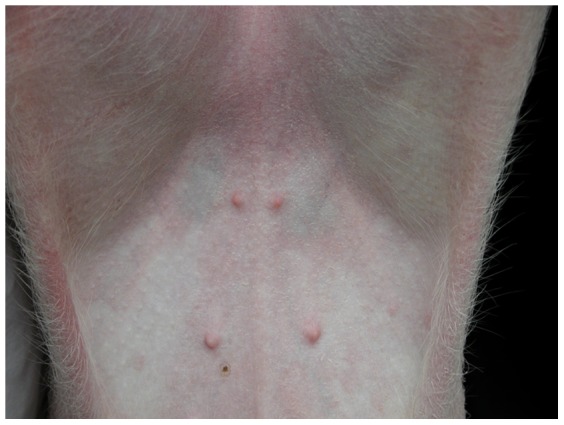
Clinical signs caused by P1 infection were usually visible as tan-to-purple inguinal lymph nodes.

Mild clinical signs of inoculated pigs were observed in the majority of the pigs in the P1 DNA-transfected Group 1, including paleness of skin and tan-to-purple inguinal lymph nodes (Figure 5). Two of 10 pigs showed mild dyspnea from 10 dpi to 16 dpi. In contrast, all control pigs in Group 2 remained clinically normal from 0 dpi to 35 dpi.

### Gross lesions

**Figure 6:**
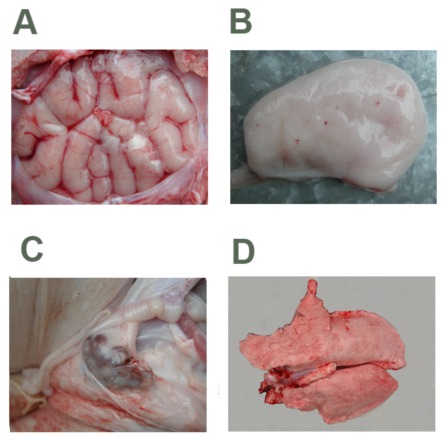
Gross findings from the pigs inoculated by the intralymphoid route with P1 DNA and necropsied at 35 dpi. (A) Cerebral edema. (B) Bladder mucous membrane bleeding. (C) Haemorrhage of the superficial inguinal lymph nodes. (D) Shrinking right lung.

Most of the pigs in the inoculated group had similar gross lesions confined to the brain, lungs, bladder, and lymph nodes (Figure 6). At necropsy, the most conspicuous changes were encephalemia (Figure 6A), haemorrhage of the bladder mucosa (Figure 6B), haemorrhage of the superficial inguinal lymph nodes (Figure 6C), and lung atrophy caused by pulmonary consolidation and even haemorrhage (Figure 6D). No gross lesions were observed in the control pigs at necropsy.

### Histopathological lesions

**Figure 7:**
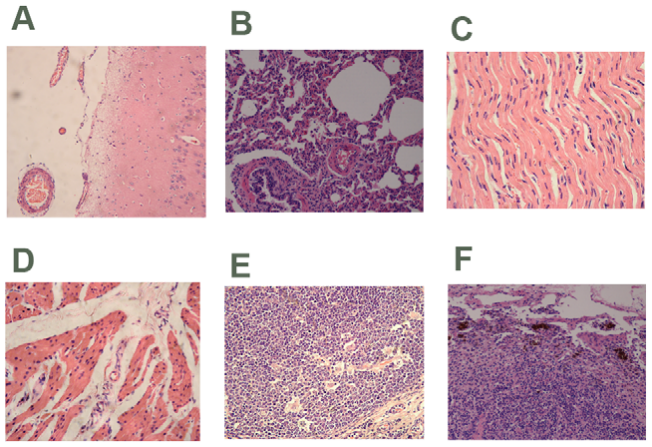
Histological patterns in different tissues of the pigs. (A) Brain of a P1-inoculated pig. Note subarachnoid arterioles and capillaries telangiectasias with a large number of red blood cells. (B) Microscopic section of a pig lung. Note interstitial pneumonia characterized by greatly thickened interlobular septum and alveolar septal thickening, a small number of alveolar septum fractures, some expansion of the alveoli filled with red blood cells. (C) Myocardial cells showed atrophy of a mild degree. (D) Note swelling of the bladder muscle cells (E) Histiocytic hyperplasia of follicles in the tonsils of the inoculated pigs. (F) Haemorrhage of the inguinal lymph nodes observed in P1 plasmid DNA transfected pigs characterized as erythrocyte infiltration and haemosiderin deposition in the expanding medullary sinus. HE staining was used for all panels. Magnification. ×40.

Pigs from the P1 plasmid DNA-transfected groups had similar lesions in brain, lung, heart, bladder, and lymphoid tissues (tonsil, and lymph nodes; Figure 7). Brain lesions were observed in 6 of 10 (Nos. 1, 2, 3, 5, 7 and 9) pigs from the inoculated groups and were characterized as moderate-to-severe arteriectasis and telangiectasia of the cavitas subarachnoidealis (Figure 7A). Lung lesions were observed in 8 out of 10 inoculated pigs (Nos. 1, 2, 3, 4, 5, 7, 8 and 9) and consisted of interstitial pneumonia characterized by multifocal to diffuse fibroblasts and lymphoid cell infiltration of the bronchus-associated lymphoid tissue, peribronchial and peribronchiolar interstitial tissue, and alveolar septa (Figure 7B). Mild atrophy of cardiac muscle cells was also observed in 5 out of 10 inoculated pigs (Nos. 1, 2, 3, 6 amd 10), which was characterized as wavilness myocardial cells (Figure 7C). Swelling of the bladder muscle cells was observed in 8 of 10 inoculated pigs (Nos. 1, 2, 3, 4, 5, 7, 8 and 9) and the nuclei of the bladder muscle cells were spindle to round in shape (Figure 7D). Mild-to-moderate histiocytic hyperplasias of the follicles were observed in the tonsils of 6 of 10 of the inoculated pigs (Nos. 1, 2, 3, 5, 7 and 9; Figure 7E). Haemorrhage of the inguinal lymph nodes was observed in 9 of 10 pigs (Nos. 1, 2, 3, 5, 6, 7, 8, 9 and 10) in the P1 plasmid DNA transfected group, which was characterized as erythrocyte infiltration and haemosiderin deposition in an expanding medullary sinus (Figure 7F). No remarkable lesions were observed in other tissues. Striking microscopic lesions were not observed in any tissues of all control pigs.

### Tissue distribution

To detect the tissue distribution of P1, P1 DNA was detected by PCR assay. The results showed that the heart (Nos. 1, 2, 3, 6 and 10), brain (Nos. 1, 2, 3, 5, 7 and 9), lungs (Nos. 1, 2, 3, 4, 5, 7, 8 amd 9), livers (Nos. 1, 2 and 3), testicles/ovaries (Nos. 1, 2, 3, 5, 7 and 9), bladder (Nos. 1, 2, 3, 5, 6, 7, 8 and 9), and pancreas (Nos. 1, 2, 3, 4, 7, 8, 9 and 10) of some of the inoculated pigs necropsied at 35 dpi were positive for P1 DNA (Table 2). All tissues from the control pigs were negative for P1 DNA.

**Table 2:** Detection of tissue distribution of P1 by PCR in infected and control pigs.

Group Inoculum Route of inoculation			heart liver spleen lung kidney pancreas brain gonads bladder tonsil inguinal mesentery lymph node lymph node											
1	P1DNA	Intralymphoid	5/10	3/10	0/10	8/10	0/10	8/10	6/10	6/10	8/10	0/10	0/10	0/10
2	pSK vector	Intralymphoid	0/10	0/10	0/10	0/10	0/10	0/10	0/10	0/10	0/10	0/10	0/10	0/10

**Figure 8:**
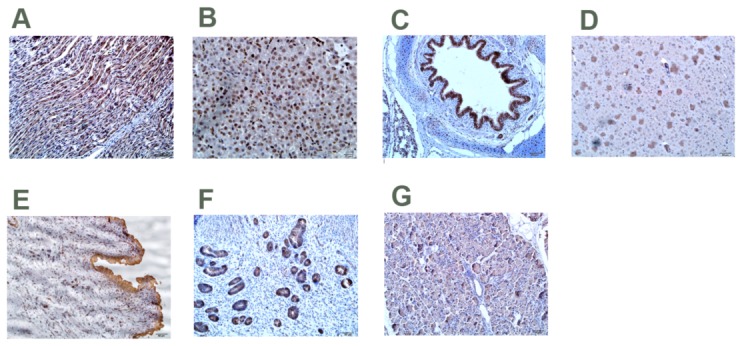
Immunohistochemical staining of the tissues of pigs with P1 infection. Hematoxylin counterstained. (A) Heart; (B) Liver; (C). Lung; (D) Brain; (E) Bladder; (F) Endometria; (G) Pancreas.

P1 antigen was also detected in the heart (Nos. 1, 2, 3, 6 and 10), brain (Nos. 1, 2, 3, 5, 7 and 9), lungs (Nos. 1, 2, 3, 4, 5, 7, 8 and 9), livers (Nos. 1, 2 and 3), testicles/ovaries(Nos. 1, 2, 3, 5, 7 and 9), bladder (Nos. 1, 2, 3, 5, 6, 7, 8 and 9), and pancreas (Nos. 1, 2, 3, 4, 7, 8, 9 and 10) by IHC. Positive cells contained dark brown reaction product for P1. P1 antigen was observed both in the cytoplasm and in the nucleus (Figure 8).

Specifically, P1 antigen was located almost exclusively in the cytoplasm of myocardial cells (Figure 8A). On the contrary, the nuclei of hepatocytes were P1-infected in the liver (Figure 8B). Intense P1-positive cells were found in the alveolar epithelial cells and bronchial epithelial cells, which were usually localized in both the cytoplasm and nucleus (Figure 8C). The brain was most consistently positive. The predominant specific staining for P1 was most often seen in nerve cells within the cytoplasm and nucleus (Figure 8D). P1 antigen was most readily detected in bladder epithelium cells and bladder muscle cells (Figure 8E). P1 antigen was present in the cytoplasm of endometrial epithelial cells (Figure 8F). The distribution of positive cells in the pancreas was very patchy. Positive signal for P1 was detected in pancreatic acinar cells and pancreas duct epithelial cells (Figure 8G). Sections from the control pigs lacked positive staining for P1 by immunohistochemistry techniques (data not shown).

## Discussion

PMWS is a multifactorial disease. Experimental inoculation of piglets with PCV2 alone indicated that PCV2 is the causative agent of PMWS [1], [2], [6], [9], [49]–[51]. However, it appears that the severity of PMWS can be increased when PCV2 is co-infected with other agents or immuonstimulated [52]. We occasionally discovered a novel agent P1 from the serum samples on diagnosis of an unexpected PMWS outcome by PCR of PCV2 DNA, which possesses a circular genomic DNA highly homologous in sequence with ORF2 of PCV2 [47], [48].

As P1 has first been identified only as a PCR product, PCR is prone to produce artifacts, it is vital to demonstrate that a P1 molecule exits. The subsequent studies confirm P1 contains a circular single-stranded DNA molecule with a size of 648 [53]. This study positively confirmed the P1 is a small non-enveloped virus by EM except its genomic DNA PCR results. The existence of self-replicating subgenomic molecules have already been demonstrated in PCV1, Mankertz et al reported four smaller subgenomic DNA molecules containing the PCV1 replication origin from the defective interfering particle that represented different defined regions of the PCV viral genome [54], and we have also reported four similar rearranged PCV2 [55]. Unlike all the above mentioned DNA molecules, P1 not only contains the insertion of the exogenous sequence, but does not share the replication origin with the parental PCV2. P1 should be considered as a result of PCV2 recombined with other organisms rather than rearranged.

To date, little is known about the biological nature of P1. However, experimental infection of pigs with the clinical samples containing P1 agent would hamper a confirmative characterization of the clinical disease and pathologic lesions solely attributable to P1 infection in the case of the presence of other known and unknown swine agents such as PCV2. Here, the study of infection by direct injection of cloned P1 genomic DNA into the lymph nodes of pigs has some advantages. Firstly, the replication and pathogenesis of P1 can be studied *in vivo* without producing infectious virus stocks by propagating P1 in cell cultures. In fact, serial cell culture passages might induce some P1 variants. Secondly, the cloned P1 plasmid DNA can be easily quantified with a spectrophotometer for pig infection. Whereas P1 virus quantification requires an indirect fluorescent antibody test or immunocytochemical staining, they are expensive and technically-demanding. More importantly, direct injection of pigs with cloned P1 plasmid DNA avoids the possible contamination of sera or tissue homogenate inocula by other endogenous swine agents.

The genomes of PCV have two major ORFs. The Rep protein encoded by the largest ORF1 is necessary to initiate replication of PCV [56], and the Cap protein encoded by ORF2 is the major structural protein of PCV. Most PCR assay primers basically target ORF2 at PCV2 diagnosis. As the sequence of P1 shared high identity with the ORF2 of PCV2, we successfully detected the load of P1 in infected PK15 cells by real-time PCR assay, as published previously when detecting PCV2 with primers targeting the ORF2 of PCV2. A more reliable PCR assay should target the ORF1 region or the complete genome of PCV2 after the emergence of P1 and the rearranged PCV2.

Although P1 has significant homology to ORF2 of PCV2, a cross-reaction should have been observed by the ORF2-based ELISA, seroconversion did not occur during the whole experimental process. In fact, ORF1 protein of P1 shared relatively low homology with PCV2 for ORF2 as a nucleotide deletion with ORF1 of P1, which might result no or low cross-reaction between P1 serum and the ELISA reaction for PCV2.

We selected the Landrace pigs for this study based on a predisposition of the Landrace pigs to PCV2-induced disease and lesions [57]. The following additional *in vivo* experiment with Guangxi Bama miniature pigs was also successful. Moreover, the phenomenon of pale skin was more easily observed in contrast to the Landrace pigs (data not shown). Pigs can be infected *via* the intra-inguinal lymph node route of inoculation with P1 molecular DNA clones (rpSK-2P1) resembling those *via* the intra-iliac lymph node route with the cloned PCV2 plasmid DNA [41].

The PMWS-like clinical signs, gross lesions, histopathological lesions, and virus distribution uniquely attributable to P1 infection were clearly characterized by the use of the rpSK-2P1. Viremia was detected in the majority of the inoculated pigs (Table 1). Similarly, the majority of experimental pigs showed clinical signs and pathologic lesions. P1 DNA was also detected by PCR and immunohistochemistry for expression of P1 proteins in various tissues and organs in inoculated pigs. Gross lesions were restricted to the brain, lungs, bladder and lymph nodes. Histopathological lesions in the brain, lung, heart, pancreas and lymphoid tissues could be detected. The pathologic lesions caused by P1 molecular DNA clones are different from those reproduced with the PCV2 molecular DNA clone; e.g., the latter were characterized by systemically enlarged, tan lymph nodes, and lungs that failed to collapse [27], whereas the former were characterized by encephalemia, haemorrhage of the bladder mucosa, haemorrhage of the superficial inguinal lymph nodes, lung atrophy and haemorrhage.

Despite more than one decade of research, many questions remain unanswered about the aetiology of PMWS because of the disease complexity. The difficulties in reproducing PMWS using PCV2 alone have resulted in a surmise that PMWS may be caused by an unknown pathogen termed agent “X”. This study provides important clues for a better understanding of PCV and PMWS. Similar to PMWS by PCV2 infection, clinical signs of the pig disease caused by P1 mainly included pale skin, but there is a big difference between P1 and PCV2 in the anatomic lesion, virus distribution in tissues, and histopathological changes. Therefore, pig diseases caused by P1 and by PCV2 should be considered as different diseases. In the current study, to our knowledge, porcine circovirus-like agent P1 with the smallest genome was identified for the first time in porcine samples and may be a new porcine circovirus member. Of course, the classification and nomenclature of P1 would be finalized by the International Committee on Taxonomy of Viruses (ICTV). Further study is needed to explore its replication, host-P1 interactions, the precise evolutionary relationships among PCV, and clinical significance. In addition, further investigations are needed to confirm that the clinical signs and pathologic lesions in the pigs injected with P1 molecular DNA clones are similar to those of pigs naturally affected by the P1 virus.

In summary, we report here for the first time that the cloned P1 genomic DNA is infectious when directly injected into the superficial inguinal lymph nodes of conventional pigs. The infectious agent P1 is one novel pathogen for pigs.

## Materials and Methods

### Ethics statement

This study was performed in strict accordance with the guidelines of Jiangsu Province Animal Regulations (Government Decree No 45). The protocol was approved by the Committee on the Ethics of Animal Experiments of the Institute of Veterinary Medicine, Jiangsu Academy of Agricultural Sciences (JAAS No 20100604).

### Molecular cloning of the P1 genome

A pair of PCR primers was designed according to the P1 sequence: sense primer F (5′- TGA GGA TCC ACT AGT AAC GGC CGC -3′) and anti-sense primer R (5′- AGT GGA TCC TCA TTT AGG GTT TAA GTG -3′). This pair of primers amplifies the complete genome of P1 with an overlapping region containing the unique *Bam*HI restriction site. To isolate genomic DNA, 300 µl of digestion buffer (10 mM Tris [pH 8.5], 1 mM ethylenediaminetetraacetic acid, plus 0.5% SDS containing 200 µg/ml of proteinase K) was added to 300 µl porcine serum samples and incubated 2 h at 55°C with constant agitation. The digested samples were extracted using the standard phenol–chloroform–isoamylalcohol procedure. The extracted DNA was amplified by PCR. The amplification reaction protocol was as follows: pre-denaturation at 94°C for 5 min, followed by 35 cycles of denaturation at 94°C for 45 sec, annealing at 56°C for 45 sec, extension at 72°C for 45 sec, and a final extension at 72°C for 10 min. The PCR product of expected size was separated by gel electrophoresis and purified with an AxyPrep™ DNA Gel Extraction Kit (AXYGEN Bio, USA).

The PCR product containing the complete P1 genome was first ligated into the pMD18-T vector (TaKaRa, Dalian, China), then transformed into *Escherichia coli* DH5α competent cells. The recombinant plasmids were verified by PCR, restriction enzyme digestion and DNA sequencing. The full-length P1 genomic DNA excised from the pMD18-T vector by digestion with the *Bam*HI restriction enzyme was inserted into the pBluescript SK (pSK) vector (Stratagene) to form a molecular clone containing a single copy genome of the P1 (rpSK-P1). The ligation was conducted with T4 DNA ligase (Promega) at 22°C for 3 h, which favors sticky-end ligations to form a tandem dimer. The tandem dimers were subsequently cloned into the pSK vector (rpSK-2P1). Recombinant plasmids, rpSK-P1 and rpSK-2P1, were also confirmed by PCR, restriction enzyme digestion and DNA sequencing. The DNA concentration of the recombinant plasmids was determined spectrophotometrically.

### Cells

PK-15 cell lines were grown at 37°C in RPMI 1640 (GIBCO™, USA) supplemented with 10% heat-inactivated fetal calf serum, 100 U of penicillin G/ml, 100 µl of streptomycin/ml and 5% CO_2_. PCR or RT-PCR was used to confirm that the PK-15 cells were free of PCV1, PCV2 and mycoplasma contamination.

### Transfection

PK-15 cells seeded into 6-well culture plates were grown to approximately 85% confluency. After one wash with Opti-MEM® I medium (Invitrogen), transfection of recombinant plasmid DNA (4.0 µg) (including rpSK-P1 and rpSK-2P1) was carried out with a commercially available lipofectamine™ 2000 reagent (Invitrogen) in the presence of Opti-MEM® I medium according to the protocol supplied by the manufacturer. Simply, the DNA/lipofectamine mixture (0.5 ml) was dispensed into each culture which had been freshly rinsed with Opti-MEM® I medium. After incubation for 5 h at 37°C, the DNA/lipofectamine mixture was replaced with RPMI 1640 supplemented with 10% fetal calf serum. Untransfected PK-15 cells and PK-15 cells transfected with empty pSK vector were included as controls.

### Anti-P1 peptide antibody

The immunogenic peptide sequence (IDDFVPPEGGTNK) was selected by using a combination of prediction software programs, then synthesized by a solid phase technique, and purified by reversed-phase HPLC by Genscript Biotechnology Co., Ltd (Nanjing, China). Two 7-week-old New Zealand White rabbits (1.8 kg to 2.0 kg) were immunized subcutaneously with the 1^st^ dosage of P1 peptide after its conjugation to the KLH carrier protein with complete Freund's adjuvant followed by subsequent boosting of the 2^nd^, 3^rd^ and 4^th^ immunogen with imcomplete Freund's adjuvant at 2 weekly intervals. The rabbits were euthanized, and serum was harvested 2 weeks after the final immunization. Anti-P1 antibody titer was measured with an enzyme-linked immunosorbent assay (ELISA) using the free peptides as coating antigens. Serum from both rabbits had ELISA titers of 1∶512,000.

### Immunochemical staining

PK-15 cells seeded in 24-well culture plates were transfected with DNA. At 48 hours post-transfection, the cells were rinsed with water, fixed in a PBS solution containing 4% paraformaldehyde for 1 h, then soaked in 3% glacial acetic acid and rinsed twice in distilled water. The fixed cells were incubated with rabbit anti-P1 polyclonal serum diluted in binding buffer (0.01% Tween 20 and 0.5 M NaCl in PBS) for 1 h at room temperature, washed twice with PBS containing 0.05% Tween 20 (PBST), incubated with biotinylated goat anti-rabbit IgG (Boster, Wuhan, China) for 20 min, rinsed three times with PBST, then incubated SABC-AP reagent for 20 min and rinsed four times with PBST. Color development was carried out with BCIP/NBT. Viral antigens were stained blue-purple in this assay.

### Single growth curve analysis of virions by real-time PCR

The rescued P1 virus stock, designated virus passage 1 (F1), was generated by transfection of PK-15 cells with the P1 infectious DNA clones. The F1 P1 virus stock was then serially passaged in PK-15 cells. Virus passage 5 (F5) was used in this study. Briefly, duplicate cultures were harvested at various times from 24 h to 120 h post-infection (hpi). The medium was collected for determining free-virion titer in the culture supernatant. The cells remaining adherent to the dishes were scraped and harvested by freeze-thawing three times for determining virus titer from the intracellular P1 infected PK-15 cells. Real-time PCR was performed following the PCV2 methods as previously described [58] to determine the P1 virus loads collected at 24 hpi, 48 hpi, 72 hpi, 80 hpi, 96 hpi, 104 hpi and 120 hpi. Real-time PCR was performed in the presence of intercalating SYBR green dye (TaKaRa, Dalian, China). A standard dilution series with a known amount of pMD-18T plasmid containing a single copy of the P1 genome was run simultaneously in each reaction mixture to quantify the virus genomic copy number.

### Negative staining electron microscopy and immunoelectron microscopy

To produce purified P1 particles, passage 1 of P1 was inoculated into PK-15 cells. The infected cells harvested at 96 hpi were frozen and thawed three times, and ultracentrifuged in CsCl for 48 h at 270000 g. The product from the CsCl gradient centrifugation were negatively stained with 2% phosphotungstic acid (PTA) for 60 s. The sample was viewed using a Hitachi H7500 transmission electron microscope.

For immunoelectron microscopy, the CsCl gradient-purified P1 was incubated with 1∶200 dilution of rabbit anti-PCV2 hyperimmune serum as previously described for 1 h at 37°C and then at 4°C overnight. The mixtures were concentrated by centrifugation for 30 min at 10000 g, resuspended in PBS and examined by EM as described previously.

### Animal experimental design

To study the pathogenic potential of the P1 molecular clone (rpSK-2P1), 20 Landrace pigs aged 1 month old were randomly assigned into two groups of 10 each. Each group of pigs was housed in separate sections of the building. The pigs were free of PCV1, PCV2, porcine reproductive and respiratory syndrome virus (PRRSV), classical swine fever virus (CSFV), pseudorabies virus (PRV), porcine parvovirus (PPV) and mycoplasmas. Each pig in Group 1 was inoculated into the left superficial inguinal lymph nodes with a total of 400 µg of the recombinant P1 plasmid DNA. The 10 pigs in Group 2 were inoculated with the same dose of empty pSK vector as negative controls. The animals were monitored daily for clinical signs of disease. Serum samples were collected from all animals on 0 days, 7 days, 14 days, 21 days, 28 days and 35 days postinoculation (dpi). All pigs were euthanized at 35 dpi and gross lesions were recorded. Tissue samples of heart, liver, spleen, lung, kidney, pancreas, brain, bladder, testicles/ovaries, tonsils, and right inguinal lymph nodes were collected at necropsy for PCR analysis, immunohistochemistry (IHC), and histopathological analysis.

### PCR analysis

To measure virus P1 viremia and the distribution in pigs transfected with P1 molecular DNA clones, sera collected on different dpi and tissue samples on 35 dpi were tested for the presence of P1 DNA by PCR. The forward primer was 5′-TTCCGGGGGAACAAAGTCGTCA-3′, and the reverse primer was 5′-GGGGGGACCAACAAAATCTCT-3′. This primer pair could amplify the whole genome of P1. The reactions were run as follows: 35 cycles of denaturation at 94°C for 45 sec; annealing at 56°C for 45 sec; and extension at 72°C for 45 sec ending with a final extension step at 72°C for 10 min. The amplified products were confirmed on 1.5% agarose gels and sequenced.

### IHC

IHC protocols for the detection of P1-specific antigens were performed on all tissues collected during necropsies at 35 dpi and fixed in 4% paraformaldehyde by standard paraffin-embedding techniques. Sections (4 µm) of tissue samples from pigs were dewaxed in xylene and rehydrated through a graded series of alcohols. Endogenous peroxidase was blocked by treating sections with 3% H_2_O_2_ for 10 min at room temperature. After three washes, antigen retrieval was performed with a hot fixes method. All slides were then incubated with the polyclonal rabbit antiserum at a 1/200 dilution in PBS (pH 7.2) for 1 h at 37°C. The sections were then washed in PBS with Tween 20 and incubated for 1 h at 37°C with biotinylated goat anti-rabbit IgG. After three washes, the slides were incubated with a streptavidin-biotin-peroxidase complex (SABC) for 20 min at 37°C. After four washes for 5 min each in PBS, the sections were incubated with the enzyme substrate diaminobenzidine tetrahy-drochloride (DAB; Boster, Wuhan, China) for 10 min at room temperature, washed in running tap water and lightly counterstained with haematoxylin for 1 min at room temperature.

### Histopathological analysis

Samples of heart, liver, spleen, lung, kidney, pancreas, brain, bladder, testicles/ovaries, tonsils, and right inguinal lymph nodes were collected and fixed by immersion in 4% phosphate-buffered paraformaldehyde. Fixed samples were dehydrated, embedded in paraffin wax, and sectioned at 4 µm and then stained with hematoxylin and eosin (HE).
